# Tropical forest loss impoverishes arboreal mammal assemblages by increasing tree canopy openness

**DOI:** 10.1002/eap.2744

**Published:** 2022-11-27

**Authors:** Sabine J. Cudney‐Valenzuela, Víctor Arroyo‐Rodríguez, José C. Morante‐Filho, Tarin Toledo‐Aceves, Ellen Andresen

**Affiliations:** ^1^ Instituto de Investigaciones en Ecosistemas y Sustentabilidad, Universidad Nacional Autónoma de México Morelia Mexico; ^2^ Escuela Nacional de Estudios Superiores, Universidad Nacional Autónoma de México Mérida Mexico; ^3^ Applied Conservation Ecology Lab, Universidade Estadual de Santa Cruz Ilhéus Brazil; ^4^ Instituto de Ecología A. C. Xalapa Mexico

**Keywords:** anthropogenic landscapes, cascading effects, endangered species, landscape structure, rare species, tree canopy, vegetation structure

## Abstract

Landscape‐scale deforestation poses a major threat to global biodiversity, not only because it limits habitat availability, but also because it can drive the degradation of the remaining habitat. However, the multiple pathways by which deforestation directly and indirectly affects wildlife remain poorly understood, especially for elusive forest‐dependent species such as arboreal mammals. Using structural equation models, we assessed the direct and indirect effects of landscape forest loss on arboreal mammal assemblages in the Lacandona rainforest, Mexico. We placed camera traps in 100 canopy trees, and assessed the direct effect of forest cover and their indirect effects via changes in tree basal area and canopy openness on the abundance and diversity (i.e., species richness and exponential of Shannon entropy) of arboreal mammals. We found that forest loss had negative indirect effects on mammal richness through the increase of tree canopy openness. This could be related to the fact that canopy openness is usually inversely related to resource availability and canopy connectivity for arboreal mammals. Furthermore, independently of forest loss, the abundance and richness of arboreal mammals was positively related to tree basal area, which is typically higher in old‐growth forests. Thus, our findings suggest that arboreal mammals generally prefer old‐growth vegetation with relatively low canopy openness and high tree basal area. However, unexpectedly, forest loss was directly and positively related to the abundance and richness of mammals, probably due to a crowding effect, a reasonable possibility given the relatively short history (~40 years) of deforestation in the study region. Conversely, the Shannon diversity was not affected by the predictors we evaluated, suggesting that rare mammals (not the common species) are the ones most affected by these changes. All in all, our findings emphasize that conservation measures ought to focus on increasing forest cover in the landscape, and preventing the loss of large trees in the remaining forest patches.

## INTRODUCTION

The ongoing global agricultural and cattle ranching expansion has led to the massive conversion of native forests into highly deforested anthropogenic landscapes (Newbold et al., 2016; Taubert et al., 2018), threatening the maintenance of biodiversity and ecosystem processes (Watling et al., 2020). Diminishing forest cover at the landscape‐scale results in population declines, species' local extinctions, and the alteration of ecological communities (Fahrig, 2003, 2013; Watling et al., 2020). In addition to the direct effects on populations and communities, a decrease in landscape forest cover can trigger uncountable indirect effects by altering species' habitats (e.g., vegetation structure) in the remaining forest patches (e.g., Morante‐Filho et al., 2016, 2018; Tscharntke et al., 2012). Understanding the relative importance of such direct and indirect effects of forest cover is pivotal to thoroughly assess and mitigate the pervasive impacts of forest loss on biodiversity, especially in the humid tropics, where most terrestrial biodiversity is found (Barlow et al., 2018) and where current rates of land‐use change are the highest (Global Forest Watch, 2021).

Both deforestation and fragmentation decrease the size of forest patches in anthropogenic landscapes (Fahrig, 2003), and there is evidence that edge effects can alter vegetation structure, particularly in smaller forest patches (Arroyo‐Rodríguez & Mandujano, 2006; Laurance et al., 2006; Santos et al., 2008; Tabarelli et al., 2012). For instance, reduced forest cover in a landscape can increase the size of canopy gaps and light incidence in forest patches, which in turn decreases microhabitat suitability for shade‐tolerant trees (Reis et al., 2021). Additionally, short‐lived pioneer trees and lianas can proliferate along patch edges (Arroyo‐Rodríguez & Toledo‐Aceves, 2009; Laurance et al., 2006), while the mortality of old‐growth tree species can increase in small patches (Laurance et al., 2000). These changes in vegetation structure alter the above‐ground biomass cycles and can drive the forest toward a stable state similar to early succession through a process known as “retrogressive succession” (Santos et al., 2008; Tabarelli et al., 2008), triggering subsequent effects on biodiversity and threatening ecosystem functionality (reviewed by Tabarelli et al., 2012). Unfortunately, our understanding about these chains of ecological effects is poor, especially for highly elusive species, such as arboreal mammals.

The effects of landscape forest cover on vegetation structure can have particular impacts on arboreal mammals, since their morphological and behavioral adaptations can make them highly vulnerable to forest disturbances (Arroyo‐Rodríguez et al., 2007; Cheyne et al., 2013; Pozo‐Montuy et al., 2011). In fact, recent studies have found that arboreal mammals not only respond to patch size, but also to within‐patch vegetation characteristics (Arroyo‐Rodríguez et al., 2007; Cudney‐Valenzuela et al., 2021), and that they can be more susceptible to forest disturbance than their terrestrial counterparts (Whitworth et al., 2019). This could have important implications for forest conservation, since arboreal mammals are involved in crucial ecological processes in the upper rainforest strata, such as pollination (e.g., Ganesh & Devy, 2000), seed dispersal (e.g., Andresen et al., 2018), herbivory (e.g., Chapman et al., 2013), and pest control (Estrada et al., 2017; Kays & Allison, 2001). Thus, understanding the direct effects of landscape forest cover on arboreal mammals and its indirect effects via changes in vegetation structure is crucial for promoting adequate management strategies for preserving these vertebrates and the ecosystem processes in which they are involved.

Here we assess the direct effects of landscape forest cover and the indirect effects through changes in vegetation structure on the abundance and taxonomic diversity of arboreal mammals in the Lacandona rainforest, Mexico. To this end, we placed camera traps in 100 canopy trees in 20 forest patches and sampled arboreal mammal assemblages for 1 year. Using structural equation modeling, we assessed the effect of forest cover (exogenous predictor), tree basal area and canopy openness (endogenous predictors) on the abundance and diversity (i.e., species richness and exponential of Shannon entropy) of arboreal mammals. We used forest cover as the single exogenous landscape predictor because it is typically correlated with other landscape structure variables, such as mean patch size, patch density, edge density, and mean interpatch distance (Bascompte & Solé, 1996; Fahrig, 2003; Villard & Metzger, 2014), and it is one of the most important predictors of biodiversity patterns in human‐modified landscapes (Fahrig, 2013; Watling et al., 2020). We particularly hypothesized that, as dispersal and resource limitations are expected to be lower in landscapes with higher forest cover (reviewed by Fahrig, 2013), forest cover could be an adequate proxy of landscape suitability for arboreal mammals, being directly and positively related to mammal abundance and diversity. We also hypothesized that forest cover could have an indirect positive effect on arboreal mammals by increasing tree basal area and decreasing tree canopy openness. This is because these two vegetation variables depend on the abundance of large trees, a key source of resources (e.g., fruits, shelter) and canopy connectivity for arboreal wildlife (Chapman et al., 1992; Dupuy et al., 2012; Pinho et al., 2020) that can be negatively impacted by forest loss (Laurance et al., 2000; Melito et al., 2021).

## METHODS

### Study site

The Lacandona rainforest is situated in the eastern portion of the State of Chiapas (91°6′42.8″–90°41′8.7″ W, 16°19′17.1″–16°2′49.3″ N), and is one of the most biodiverse areas in Mexico and hence of high conservation priority (Arriaga et al., 2000). The Lacandona rainforest encompasses ~634,760 ha (de Jong et al., 2000); however, in the last four decades more than 45% of its original forest cover has been converted to other land uses, mainly annual crops, oil‐palm, rubber plantations, and cattle pastures, creating a highly fragmented landscape (Carabias et al., 2015). Mean annual temperature is 24°C (van Breugel et al., 2006), and annual rainfall ranges from 1500 to 3500 mm (Instituto Nacional de Ecología, 2000).

**FIGURE 1:**
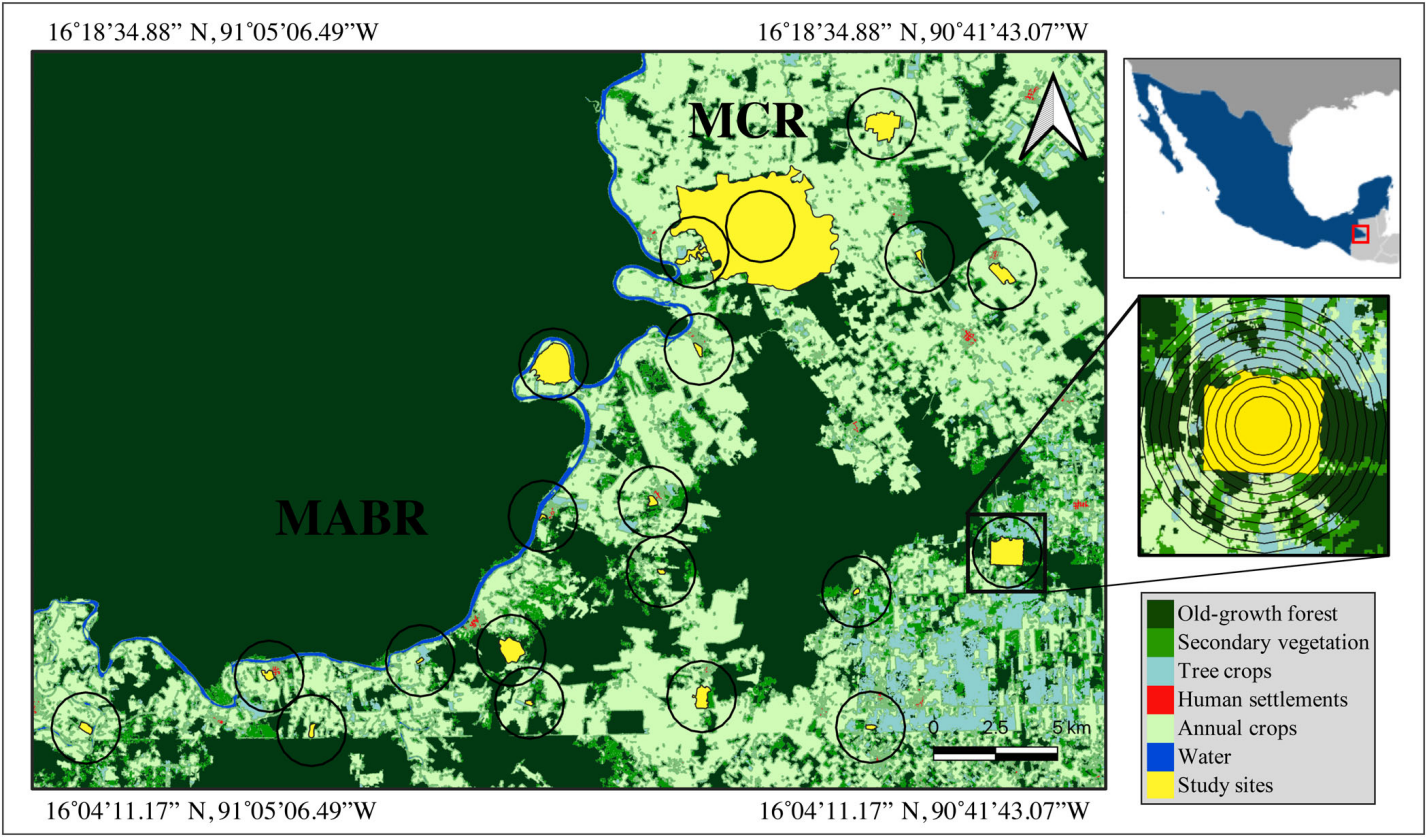
Location of the 20 forest patches (shown in yellow) used to sampling arboreal mammals in the Marqués de Comillas region (MCR), Chiapas, Mexico. The MCR is separated from the Montes Azules Biosphere Reserve (MABR) by the Lacantún River (shown in blue). The circle around each patch represents the largest buffer (1300 m radius) defining the landscapes. We also present one landscape zoomed in, showing the 10 buffers (300–1300 m radii), which were used to determine the most adequate spatial scale (scale of effect). The outline of Mexico is shown in dark blue.

Our study was carried out in the Marqués de Comillas region within the Lacandona rainforest (Figure 1), which comprises 203,999 ha of fragmented forest (Arce‐Peña et al., 2019). We selected 20 old‐growth forest patches (area ranging from 5 to 2170 ha), separated from each other by at least 2.5 km (distances measured from their geographical centers; Figure 1).

### Vegetation structure

We standardized the number of plots used in each forest patch to sample vegetation to avoid confounding the effect of patch size with the sampling area effect (i.e., the larger the sampled area, the more species and heterogeneity is expected to be found; see Fahrig, 2013). In the center of each forest patch, we established five 10 × 50 m plots separated by at least 30 m, avoiding forest edges and vegetation gaps. In these plots, we measured the diameter at breast height (dbh) of all trees with dbh ≥10 cm, and calculated the sum of tree basal area in each plot. We also estimated the percentage of canopy openness inside each plot by taking three hemispherical photographs (25 m apart) with a wide‐eye lens (Apexel 198° Fisheye Lens). The photographs were analyzed using the program Gap Light Analyzer (Frazer et al., 1999). Both tree basal area and canopy openness are considered proxies for tree biomass (Dupuy et al., 2012), which is known to increase with stand maturity (Poorter et al., 2016). Tree basal area also drives the availability of resources for arboreal mammals (Chapman et al., 1992; Pinho et al., 2020) and canopy openness is inversely related to canopy connectivity.

### Sampling of arboreal mammals

Mammal surveys are detailed elsewhere (Cudney‐Valenzuela et al., 2021; Cudney‐Valenzuela, Arroyo‐Rodríguez, Andresen, et al., 2022), but a brief overview is given here. As suggested by Fahrig (2013), sampling effort was not proportional to patch size, but standardized across landscapes with different proportions of forest cover to avoid potential confounding effects related to the so‐called “sample‐area effect.” Within the same vegetation plots, we selected five focal trees with suitable climbing conditions (branches ≥20 cm wide, preferably hardwood species) and whose architecture allowed installing a camera trap facing other main branches. In each tree, we established a single‐rope climbing system. Focal trees in the same patch were separated from each other by a distance ≥30 m. Of the five focal trees per patch, four reached the canopy (mean height ± SD = 21.8 ± 6.2 m, range = 10.2–36.6 m) and one the midstory (9.1 ± 4.7 m, 3.4–19.6 m). This allowed us to capture a greater vertical range of strata potentially used by arboreal mammals.

Within each patch, we used one camera trap (Bushnell Trophy Cam HD Aggressor Low Glow^©^) at a time, which was rotated among the five focal trees once a month, except from October to December when they remained on the same focal tree. Cameras were placed at varying heights depending on the characteristics of the focal tree (camera height of canopy and midstory trees was 15 ± 4.3 m and 2 ± 0.6 m, respectively). Cameras were continuously active from May 2018 to May 2019, and were serviced once a month (change of batteries, downloading of pictures, replacement of malfunctioning cameras). Total sampling effort was 7387 camera trap days (i.e., number of days the camera was deployed; average per patch = 369 ± 11.6 days), with 6233 active camera trap days (i.e., number of days the camera was actively recording; average per patch = 311.7 ± 19.9 days).

To increase the probability of photo‐capturing arboreal mammals inhabiting the forest patches, we used baits in the midstory trees (tuna fish, peanut butter with oatmeal, and banana). As revealed by photographs, bait was consumed during the first two nights. Since we did not provide more bait while the camera was active on that tree and no camera malfunctioned during the baited period, all sites had the same baited sampling effort. We processed all photographs with the program Digikam^©^ and extracted photograph metadata with the package “camtrapR” (Niedballa et al., 2016). We considered photo‐captures as independent events when there was at least a 24‐h interval between captures of the same species, since individuals photographed on the same day are likely the same ones (Royle et al., 2009). We identified each mammal species based on Reid's (2009) field guide. Except for the Mexican hairy porcupine (*Coendou mexicanus*) and squirrels, all other rodents were excluded from the analyses due to imprecision in identification from photographs.

We calculated each species' relative abundance index (O'Brien, 2011) by dividing the number of events for a given species by the number of days the camera was active in the forest patch, and then multiplying it by 100. This index is widely used as a proxy of mammal abundance in studies using camera traps (e.g., Benchimol & Peres, 2021; Cassano et al., 2012; Mandujano & Pérez‐Solano, 2019; Srbek‐Araujo & Chiarello, 2005). Then, we summed the relative abundance index of the species recorded in each patch as an estimate of total abundance of arboreal mammals per forest patch. We used the “entropart” package (Marcon & Hérault, 2015) to estimate species diversity using Hill numbers of order 0 (species richness, ^0^ *D*) and 1 (exponential of Shannon entropy, ^1^ *D*) (Jost, 2006). The formulas used to calculate the Hill numbers can be found elsewhere (Jost, 2006). Species richness (^0^ *D*) gives a disproportionate weight to rare species while the exponential of Shannon entropy weighs species' abundances without disproportionately favoring either rare or dominant species, and is therefore interpreted as the number of common (or typical) species in the assemblage (Jost, 2006).

### Forest cover and scale of effect

As we did not know a priori which was the best scale to assess direct and indirect forest cover effects, we followed the protocol suggested by Jackson and Fahrig (2015) to identify the scale of effect of forest cover. The analyses for calculating the scale of effect are detailed elsewhere (Cudney‐Valenzuela, Arroyo‐Rodríguez, Andresen, et al., 2022), but a brief overview is given here. First, we adopted a site‐landscape approach (sensu Brennan et al., 2002), in which response variables were measured in same‐sized sample sites (i.e., five focal trees at the center of each forest patch), and forest cover (in percentage; i.e., area covered by old‐growth forest divided by landscape size × 100) was measured within 11 circular concentric radii (300‐m to 1300‐m radius, at 100 m intervals) from the geographical center of each forest patch (Figure 1). We used recent and high‐resolution Sentinel S2 satellite images (obtained in 2016) to produce land‐cover maps of each landscape using ENVI 5.0 software, and extracted forest cover metrics using ArcGIS software with the “Patch Analyst” extension.

To identify the scale at which forest cover best predicted the response variables, i.e., the scale of effect (Jackson & Fahrig, 2015), we used generalized linear models with a Gaussian distribution error for vegetation response variables (tree basal area and canopy openness), and Poisson distribution error for abundance, species richness, and the exponential of Shannon entropy of arboreal mammals. Vegetation response variables were standardized to zero mean and unit variance using the “vegan” package for R version 3.6.0 (Oksanen et al., 2016) to make the coefficients (slope parameters) comparable since they were measured at different scales. We quantified the effect of forest cover on each response variable at each scale (1 landscape metric × 11 landscape buffers = 11 models per response variable), and used the percentage of explained deviance of each model to identify the most appropriate landscape size to assess the effect of forest cover amount on response variables (Appendix S1: Figure S1). Forest cover was significantly correlated with patch size at all selected scales of effect (Appendix S1: Table S1).

### Data analysis

We used structural equation models (SEM) to estimate the direct and indirect effects of landscape forest cover on arboreal mammal total abundance, species richness, and exponential of Shannon entropy. We tested each variable for multivariate normal distribution using Mardia's multivariate normality test (Shipley, 2016), and log transformed the variables that did not meet this criterion (i.e., forest cover at 900 m radius, and tree basal area). We tested for correlations between variables using Pearson correlation tests, and found that canopy openness was the only variable correlated with forest cover (*r* = −0.55, *p* < 0.05; Appendix S1: Table S1). However, following Cole et al. (2007), we included these two correlated variables in the model because they are part of our theoretical model and reflect features of the research design. We built three different SEM models (one for each community response: abundance, species richness, and exponential of Shannon entropy) where forest cover was included as an exogenous predictor, and tree basal area and canopy openness were considered endogenous predictors. All models were created using the “lavaan” package (Rosseel, 2012) for R software (R Core Team, 2021), and each model was composed of four variables and 20 observations. We evaluated the goodness of fit of each model using four complementary methods: a χ^2^ goodness‐of‐fit test of the difference between the observed data and hypothesized model, the Tucker‐Lewis Fit Index (TLI), the Comparative Fit Index (CFI), and the Root Mean Square Error of Approximation (RMSEA). Following Zhang et al. (2013), a satisfactory model fit was determined when we found: (a) a nonsignificant χ^2^ goodness‐of‐fit test (*p* > 0.05), (b) TLI >0.9, (c) CFI >0.9, and (d) lower 90% confidence intervals of RMSEA <0.05. All models showed satisfactory goodness of fit based on these criteria, suggesting that our conceptual model described the data adequately (Appendix S1: Table S2). We used the standardized path coefficients (β) and *p*‐values to assess the significance of individual variables within each model (Appendix S1: Table S2). The coefficient of determination (*R* ^2^) shows the variance of each endogenous variable due to the effect of the other variables.

## RESULTS

We obtained 1672 independent photo‐captures of 15 species. The most frequently recorded species were Deppe's squirrels (*Sciurus deppei*), kinkajous (*Potos flavus*), and black howler monkeys (*Alouatta pigra*), together representing 49.5% of all records. Rarely recorded species were margays (*Leopardus wiedii*), northern raccoons (*Procyon lotor*), and tayras (*Eira barbara*), together representing 0.9% of the records. On average, species were recorded in 13 of 20 sites; 11 of 15 species were present in more than half of the sites.

**FIGURE 2:**
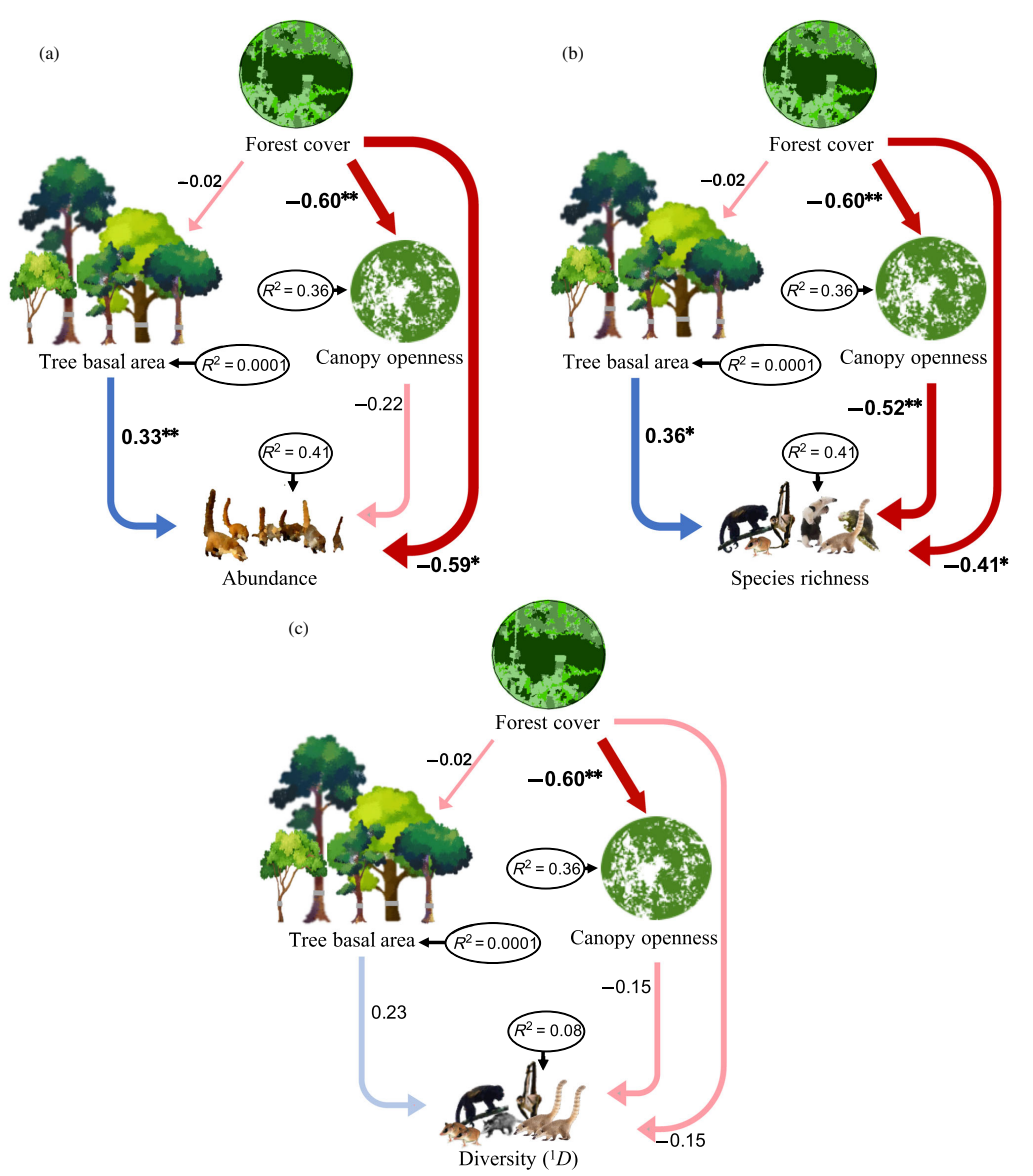
Path models of direct and indirect effects of forest cover on (a) abundance, (b) species richness, and (c) diversity (^1^ *D*; exponential of Shannon entropy). Indirect effects of forest cover occur via two vegetation structural attributes (canopy openness and tree basal area). Significant pathways are indicated with asterisks (*p < 0.05; **p < 0.01) to the right of the standardized path coefficients, while blue and red lines indicate positive and negative effects, respectively. Arrow thickness is scaled to illustrate the relative strength of the effects. The coefficient of determination (*R* ^2^) is shown within black ellipses for all response variables.

Landscape forest cover (exogenous predictor) negatively affected canopy openness in the forest patches (β = −0.60; *p* = 0.001; 36% of explained variance) but did not affect tree basal area (β = −0.02; *p* = 0.90; 0.01% of explained variance; Figure 2a; Appendix S1: Table S3). Mammal abundance in the forest patches (41% of explained variance) was not related to canopy openness (β = −0.22; *p* = 0.245), but was positively related to tree basal area (β = 0.33; *p* = 0.05) and negatively to forest cover (β = −0.59; *p* = 0.002). Species richness (^0^ *D*, 41% of explained variance) was positively related to tree basal area (β = 0.36; *p* = 0.037) and negatively related with canopy openness (β = −0.52; *p* = 0.005) and forest cover (β = −0.41; *p* = 0.029; Figure 2b; Appendix S1: Table S3). Species diversity (^1^ *D*, 8% of explained variance) was not related to canopy openness (β = −0.15; *p* = 0.532), tree basal area (β = 0.23; *p* = 0.286) nor forest cover (β = −0.15; *p* = 0.514; Figure 2c; Appendix S1: Table S3).

## DISCUSSION

Our findings reveal that landscape‐scale deforestation can impact arboreal mammals through both direct and indirect pathways, with the latter acting via changes in vegetation structure within forest patches. Particularly notable is the negative indirect effect of forest loss on mammal richness by increasing tree canopy openness, which suggests that deforestation impacts mammals by promoting habitat degradation. The abundance and richness of mammals directly increased in patches surrounded by lower forest cover, a surprising finding that could be explained by a crowding or a dilution effect (Gestich et al., 2022; Grez et al., 2004; Vallejos et al., 2020). However, independently of forest cover, species richness was positively influenced by tree basal area and negatively related to canopy openness, two vegetation structural characteristics associated with vegetation maturity. The fact that species richness responded to changes in forest cover while the Shannon diversity did not, suggests that rare species are the most affected by these changes. As discussed in the *Implications for conservation* section, these findings have important ecological and applied implications.

### Cascading effects of forest cover on arboreal mammals

Our results suggest that species richness decreases with forest loss through the increase of tree canopy openness. Other studies have also found that forest loss increases the size of canopy gaps and light incidence within forest patches, which can in turn decrease microhabitat suitability for shade‐tolerant and animal‐dispersed trees (Mascarenhas‐Lima & Mariano‐Neto, 2014; Reis et al., 2021). Deforestation can also increase the susceptibility of tree stands to wind damage (Zeng et al., 2009) and tree mortality caused by desiccation (Briant et al., 2010), which can increase tree mortality and gap formation within the remaining forest patches. Thus, following previous studies (see Melito et al., 2021), preventing further tree mortality within forest patches is a matter of maintaining forest cover in the surrounding landscape, as previously known in terms of forest patch size (Laurance et al., 2000).

The negative relationship between arboreal mammal richness and canopy openness aligns with multiple studies that show canopy connectivity to be important for these mammals (Di Bitetti et al., 2000; Palminteri et al., 2012). Arboreal mammals have evolved morphological adaptations that make them dependent on canopy structure for movements in the upper forest strata (Fleagle & Lieberman, 2015), which makes them particularly sensitive to alterations in canopy connectivity (Cheyne et al., 2013; Pozo‐Montuy et al., 2011). The increase of canopy openness in patches embedded in more deforested landscapes is associated with tree mortality and damage (Clark et al., 2004; Ibanez et al., 2018), which can also result in reduced availability of and/or access to food resources and shelters.

Contrary to our predictions, forest cover did not have a significant effect on total tree basal area, which could be related to our sampling design. We expected to find a proliferation of short‐lived pioneer trees as a result of edge effects. But, since edge effects diminish with increasing distance from the edge, placing our sampling plots at the center of the forest patches could have reduced our ability to detect such effect. Moreover, the fact that our study region has a relatively short history of land‐use change (<40 years; de Vos, 2002, Global Forest Watch, 2021) could also imply that changes in vegetation structure are not yet conspicuous, as previously demonstrated by Hernández‐Ruedas et al. (2014) in the study region.

However, the fact that, independently of forest cover, we found more individuals belonging to more species in patches with higher total tree basal area, underscores the importance of this vegetation attribute for this forest‐dependent faunal group. This is not surprising, as other studies have shown that tree basal area is a major driver of forest patch occupancy by howler monkeys (Arroyo‐Rodríguez et al., 2007) and spider monkeys (Urquiza‐Haas et al., 2009), likely because it can reflect the availability of resources (e.g., food, shelter, support) for arboreal mammals (Chapman et al., 1992; Pinho et al., 2020). Tree basal area is also positively associated with stand maturity (Dupuy et al., 2012), a local feature strongly related to invertebrate density and diversity (Jeffries et al., 2006). This can be particularly important for insectivorous and omnivorous arboreal mammals—such as anteaters and the four species of opossums found on this study—given that stands with greater basal area could be offering more diverse and abundant food resources (Pinho et al., 2020). Therefore, forest remnants composed of large old trees could have higher quality and quantity of resources, potentially leading to the relaxation of interspecific competition (Asensio et al., 2007), and thus promoting species coexistence.

### Direct effect of forest cover on arboreal mammal abundance and richness

Our findings showed that forest cover also had direct effects on the abundance and richness of arboreal mammals but, unexpectedly, these effects were negative. These results may be explained by two related, not mutually exclusive, phenomena. First, high forest cover in the landscape could decrease the chances of individuals visiting the trees (and branches) where the cameras were placed, since more forest cover offers more foraging area, creating a dilution effect. Second, it is possible that our study patches may be experiencing a crowding effect when found in highly deforested landscapes. In particular, population abundance could be higher due to increased dispersal of individuals into the focal patches from the surrounding and recently deforested areas (Gestich et al., 2022; Grez et al., 2004; Vallejos et al., 2020). This is a plausible explanation of the negative association between forest cover and mammal abundance and richness because the remaining forest patches in more deforested landscapes are known to act as temporal refuges (Schmiegelow et al., 1997), which can increase photo‐captures, even when the patches are not permanently inhabited. Such a potential crowding effect can be facilitated by the lack of top‐predators (e.g., *Harpia harpyja*, *Panthera onca*) and their regulatory effect on mammal populations, as these predators are usually among the first animals to disappear from forest patches (e.g., Benchimol & Peres, 2021). However, it is necessary to be careful with this result as individuals in more deforested landscapes may not survive in the long term. As argued by Gestich et al. (2022) and others (e.g., Arroyo‐Rodríguez & Dias, 2010; Gabriel et al., 2018; Marsh et al., 2013), increased densities could expose species to high levels of intraspecific and interspecific competition, as well as increased physiological stress disease incidence. Therefore, additional long‐term monitoring studies are needed to test this hypothetic scenario.

### Response of rare vs common species to vegetation changes

The fact that species richness directly and indirectly responded to forest cover while the exponential of Shannon entropy did not, suggests that rare species (not the common ones) are the most affected by vegetation changes at the local and landscape scales. Rare species tend to have small populations, lower densities, and can be more specialized than common species (Doherty & Harcourt, 2004; Gaston, 1997), which could further increase their extinction risk in fragmented landscapes. In fact, of the five rarest species in this study, four are listed as locally threatened: margays (*Leopardus wiedii*), tayras (*Eira barbara*), coatis (*Nasua narica*) and Northern tamanduas (*Tamandua mexicana*) (SEMARNAT, 2010). Moreover, after a year of sampling, our study did not find pigmy silky anteaters (*Cyclopes didactylus*)—a known rare mammal of the region—which could suggest that populations from this species are being reduced in fragmented landscapes. Conversely, the lack of response of the common species could be explained by their tolerance to habitat disturbance, as has been documented for four of the five most common species of this study: Deppe's squirrels (*Sciurus deppei*; Koprowski et al., 2016), kinkajous (*Potos flavus*; Keeley et al., 2017), wooly opossums (*Caluromys derbianus*; Solari & Lew, 2015), and mouse opossums (*Marmosa mexicana*; Martin, 2016). This suggests that there is a differential response within arboreal mammal assemblages to changes in forest loss and vegetation structure, so that future research should consider studying this group of mammals according to guild and/or degree of specialization.

## CONSERVATION IMPLICATIONS

Our study shows that landscape‐scale deforestation in the Lacandona rainforest has negative indirect effects on arboreal mammals through the increase of canopy openness, stressing that conservation actions must not only be focused on the extent of remaining habitat but on its quality as well (Cudney‐Valenzuela et al., 2021). Our results also align with recent evidence suggesting that more individuals and species of arboreal mammals can be found in more deforested landscapes due to a crowding effect (Cudney‐Valenzuela et al., 2021; Gestich et al., 2022).

Our study suggests that to maintain rainforest arboreal mammal assemblages in human‐modified landscapes, conservation measures should be focused on increasing forest cover in the landscape, while maintaining high tree basal area and reducing canopy openness within forest fragments. Increasing forest cover in the landscape can be achieved by passive and active forest restoration. Conversely, to maintain basal area and reduce canopy openness, it is particularly important to prevent the loss of large old trees within forest patches. To achieve the latter, it is important to minimize edge effects and selective logging. In fact, selective logging has been found to cause the decline of large‐sized mammals (Jamhuri et al., 2018), reduce mammal richness (Burivalova et al., 2014), and increase mammal stress responses, especially in threatened species (Messina et al., 2018), emphasizing the importance of phasing‐down selective logging in order to conserve arboreal mammals. Finally, as suggested in previous studies (Balbuena et al., 2019; Chan et al., 2020; Gregory et al., 2017), we would recommend promoting canopy connectivity inside forest patches with large canopy gaps, by installing canopy rope bridges, which are artificial rope structures that connect trees, mimicking lianas and allowing arboreal mammals to move in the canopy.

## AUTHOR CONTRIBUTIONS

Sabine J. Cudney‐Valenzuela and Víctor Arroyo‐Rodríguez developed the idea of the study, with support from Ellen Andresen and Tarin Toledo‐Aceves. Sabine J. Cudney‐Valenzuela collected and analyzed the data with guidance from Víctor Arroyo‐Rodríguez and José C. Morante‐Filho. All authors made substantial contributions to the intellectual content, interpretation, and editing of the manuscript.

## CONFLICT OF INTEREST

The authors declare no conflict of interest.

## Supporting information


Appendix S1

## Data Availability

Data and code (Cudney‐Valenzuela, Arroyo‐Rodríguez, Morante‐Filho, et al., 2022) are available from Figshare: https://doi.org/10.6084/m9.figshare.19319894.v3.
