# Supplementary material for: Tropical forest loss impoverishes arboreal mammal assemblages by increasing tree canopy openness
Source: Ecol Appl. 2022 Nov 27;33(1):e2744. doi: 10.1002/eap.2744 (PMC10078566; doi:10.1002/eap.2744)
Supplement: Supplementary file 1 — Appendix S1 [file EAP-33-0-s001.pdf]

Appendix S1

Cudney-Valenzuela et al. Tropical forest loss impoverishes arboreal mammal assemblages by increasing tree canopy openness. *Ecological Applications*

Table S1. Correlation matrix between predictor and explanatory variables using Pearson's correlation test. Only the correlation values at the scale of effect are shown for landscape-response variable associations. Significant correlations are highlighted in bold (*p<0.05, **p<0.01).

	Tree basal area	Canopy openness	Abundance	Species richness	Shannon diversity	Patch size
Forest cover (300 m radius)	0.17			-0.21	-0.1	**0.66****
Forest cover (400 m radius)			**-0.52***			**0.75****
Forest cover (900 m radius)		**-0.55****				**0.63****
Tree basal area		-0.30	0.43	**0.46***	0.26	
Canopy openness			0.03	0.43	0.14	

Table S2. Results of the adjustment coefficients of all models. For each model, we showed the degrees of freedom (DF), test statistic (minimum function chi-square, χ^2), model P-value, comparative fit index (CFI), Tucker-Lewis fit index (TLI), and root mean square error of approximation (RMSEA).

Model	DF	χ^2	P-value	CFI	TLI	RMSEA
Abundance	4	6.745	0.456	1.00	1.028	0.00
Species richness	4	2.330	0.675	1.00	1.259	0.00
Shannon diversity	4	2.896	0.575	1.00	1.513	0.00

Table S3. Test statistics for each model. We showed the unstandardized path coefficients (Coef.), the related standard error (SE), Z-value, P-value and standardized path coefficients (β). The significant paths are highlighted in bold.

Regressions	Coef	SE	Z	*P*	ß
Tree basal area ~ Forest cover	-0.54	0.66	-0.082	0.935	-0.018
Canopy openness ~ Forest cover	**-1.019**	**0.303**	**-3.361**	**0.001**	**-0.601**
**Abundance** ~ Forest cover	**-21.725**	**6.942**	**-3.129**	**0.002**	**-0.599**
Tree basal area	**3.729**	**1.926**	**1.936**	**0.053**	**0.333**
Canopy openness	-1.971	1.697	-1.161	0.245	-0.222
**Species richness** ~ Forest cover	**-1.927**	**0.881**	**-2.187**	**0.029**	**-0.407**
Tree basal area	**0.571**	**0.274**	**2.086**	**0.037**	**0.359**
Canopy openness	**-0.659**	**0.234**	**-2.810**	**0.005**	**-0.523**
**Shannon diversity** ~ Forest cover	-0.462	0.707	-0.653	0.514	-0.151
Tree basal area	0.234	0.220	1.066	0.286	0.229
Canopy openness	-0.118	0.188	-0.626	0.532	-0.145

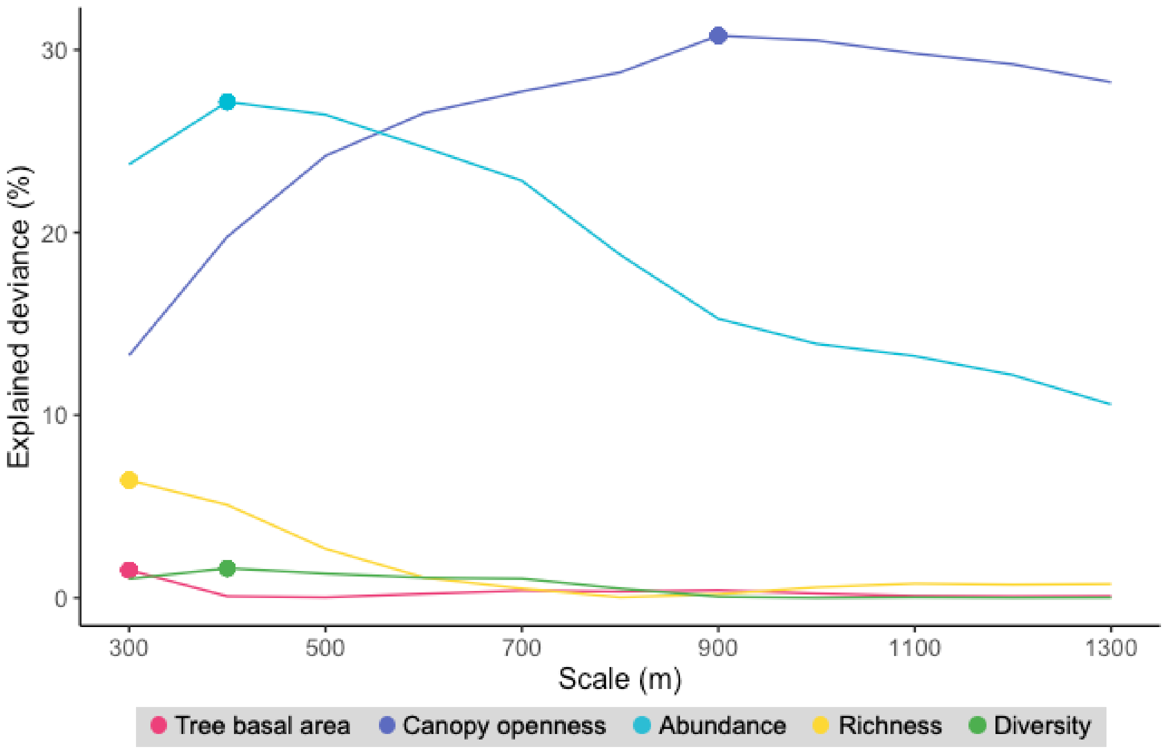

Figure S1. Selection of the best spatial scale (i.e. scale of effect) used to indicate the relationship between forest cover and each endogenous predictor. Coloured dots indicate the scale of effect.
